# A personalized three-dimensional printed knee splint for mechanical mitigation of canine knee osteoarthritis and adjunctive nonsteroidal anti-inflammatory drug therapy

**DOI:** 10.3389/fvets.2026.1938731

**Published:** 2026-10-26

**Authors:** Xiao-tong Li, Zi-han Xiong, Jia-ming Ye, Wei-tao Qiu, Jing-feng Sun, Jia-le Gu, Yue-Yin, Zhan-lun Ou, Shao-chuan Li

**Affiliations:** 1 Teaching Animal Hospital, College of Veterinary Medicine of South China Agricultural University, Guangzhou, China; 2 Foshan Zhengheng Biotechnology Co., Ltd., Foshan, China; 3 Guangzhou Liming Biotechnology Co., Ltd., Guangzhou, China

**Keywords:** 3D printing, canine osteoarthritis, finite element analysis, monosodium iodoacetate, nonsteroidal anti-inflammatory drugs, personalized knee splint, quality of life

## Abstract

**Introduction:**

Canine knee osteoarthritis (OA) involves pain, inflammation, altered joint loading, and restricted motor function. Because nonsteroidal anti-inflammatory drugs (NSAIDs) do not directly correct abnormal joint mechanics, this study evaluated a personalized three-dimensional (3D)-printed polylactic acid knee splint, used alone or with short-term NSAID treatment, in a monosodium iodoacetate-induced beagle knee OA model.

**Methods:**

Twelve dogs were assigned to four groups (*n* = 3 per group): OA without continued treatment (OA), splint alone (OA/Spt), NSAIDs alone (OA/NSAIDs), and combined NSAIDs plus splint treatment (OA/NSAIDs/Spt). *In vivo* outcomes included static hindlimb weight-bearing symmetry, knee joint range of motion (ROM), and Liverpool Osteoarthritis in Dogs (LOAD), The Canine Brief Pain Inventory (CBPI), and The CanineOrthopedic Index (COI) pain and function scores. Finite element analysis using representative healthy and OA knee models was used as descriptive, model-based mechanical support.

**Results:**

The splint remained belowthe polylactic acid (PLA) yield threshold under simulated loading and reduced several model-predicted mechanical indices in the OA model, including peak stress, equivalent elastic strain, femoral condylar contact pressure, and cartilage total deformation. *In vivo*, splint treatment alone was associated with better short-term functional outcomes than no continued treatment, whereas the OA/NSAIDs/Spt group showed the greatest overall improvement in weight-bearing symmetry, ROM, and clinical scores.

**Discussion:**

Because no formal interaction analysis was performed, the combined-treatment findings should be interpreted as broader short-term functional benefit rather than evidence of an interaction effect. These preliminary results support personalized 3D-printed splinting as a potential mechanical adjunct to NSAID therapy for canine knee OA.

## 1 Introduction

Canine osteoarthritis (OA) is a common chronic joint disease associated with pain, impaired mobility, altered limb loading, and progressive loss of joint function (1, 2). A recent prospective study reported radiographic appendicular OA in 39.8% of dogs aged 8 months to 4 years, with 23.6% meeting criteria for clinical OA with at least mild pain (3). In affected dogs, reduced weight-bearing, decreased mobility, and limited joint function are clinically important outcomes in addition to pain itself (4). Treatment strategies should therefore address both symptoms and functional loading.

Canine OA management is still largely pharmacological. Nonsteroidal anti-inflammatory drugs (NSAIDs) remain widely used because they reduce pain and inflammation and can improve mobility (5, 6). However, they do not directly correct OA-related abnormal joint loading, and long-term use may cause gastrointestinal or systemic adverse effects (7). These limitations support investigation of adjunctive non-pharmacological approaches that can improve mechanical support and functional status (8).

In this study, biomechanical intervention refers to a physical or structural approach intended to improve affected-limb function by providing external support, limiting abnormal motion, or redistributing joint loading. Evidence for such interventions in canine OA remains limited, and it is unclear whether mechanical support adds functional benefit to drug therapy (9, 10). This question is clinically relevant because NSAIDs primarily target pain and inflammation, whereas a splint may address joint loading through external support.

Three-dimensional (3D) printing enables patient-specific orthopedic devices and anatomical models (11, 12). For external orthotic support, device geometry and material properties influence fit, stability, and function (13). A splint produced from surface scanning and digital modeling may better match canine limb anatomy than a non-personalized device, making it suitable for evaluating mechanical adjunctive treatment in canine knee OA (14).

Finite element analysis (FEA) can estimate load distribution in complex anatomical structures and is useful for model-based biomechanical assessment (15, 16). Here, FEA was used to evaluate the potential effects of the splint on load distribution in representative healthy and OA knee models. The *in vivo* experiment was used to evaluate short-term functional outcomes.

Against this background, the objective of this study was to design a personalized 3D-printed polylactic acid (PLA) knee splint and to evaluate its potential biomechanical unloading effect using finite element analysis. In an *in vivo* beagle model of knee OA, we further compared short-term functional outcomes among dogs receiving no continued treatment after the common analgesic period (OA), splint treatment alone (OA/Spt), NSAID treatment alone (OA/NSAIDs), or combined NSAID plus splint treatment (OA/NSAIDs/Spt). We hypothesized that the splint would improve OA-related weight-bearing asymmetry and mechanical dysfunction and that combined treatment would provide broader short-term functional benefits than either intervention alone.

## 2 Materials and methods

### 2.1 Experimental animals and grouping

The experimental beagles were obtained from the Guangdong Beagle Dog Germplasm Resource Research and Development Center, Guangzhou General Pharmaceutical Research Institute Co., Ltd. (production license No. SCXK [Yue] 202300071). The 12 experimental beagles weighed 11.0 ± 1.4 kg on average. All dogs were 12 months old, with equal numbers of males and females. Before enrollment, all dogs were judged to be healthy by clinical examination, with no obvious orthopedic or systemic disease, and had complete vaccination records (rabies vaccine batch No. E138713A; combined vaccine batch No. 735284A). The study protocol was approved by the Animal Welfare and Ethics Committee (approval No. 2026005), and all animal experiments were performed at Guangzhou Liming Biotechnology Co., Ltd.

Dogs were ranked by body weight and divided into three blocks of four animals. Within each block, one dog was randomly assigned to each of four experimental groups, giving three dogs per group: OA without continued treatment (OA), splint alone (OA/Spt), NSAIDs alone (OA/NSAIDs), and combined NSAID plus splint treatment (OA/NSAIDs/Spt).

After a 1-week acclimation period, baseline static weight-bearing, range-of-motion, and clinical score data were collected before OA induction. Finite element modeling was performed using cone-beam computed tomography (CBCT) data from one healthy beagle and one representative dog from the OA/Spt group. These imaging data were used only to construct computational models and were not included as additional animals in the group-level statistical analysis.

### 2.2 Main materials, equipment, and assessment tools

#### 2.2.1 Agents used for OA induction and treatment

Monosodium iodoacetate (MIA) was purchased from Shanghai E Chemical Technology Co., Ltd. (catalog No. R014800). Following the modeling protocol of Pomonis et al. (17). MIA was administered as 100 mg in 0.5 mL saline by intra-articular injection into the knee joint. Injectable meloxicam was obtained from Qilu Animal Health Products Co., Ltd. (veterinary drug approval No. 150252467; product model No. E136H004) and administered subcutaneously at 0.1 mg/kg, with a doubled first dose of 0.2 mg/kg (Figure 1Aa).

**Figure 1:**
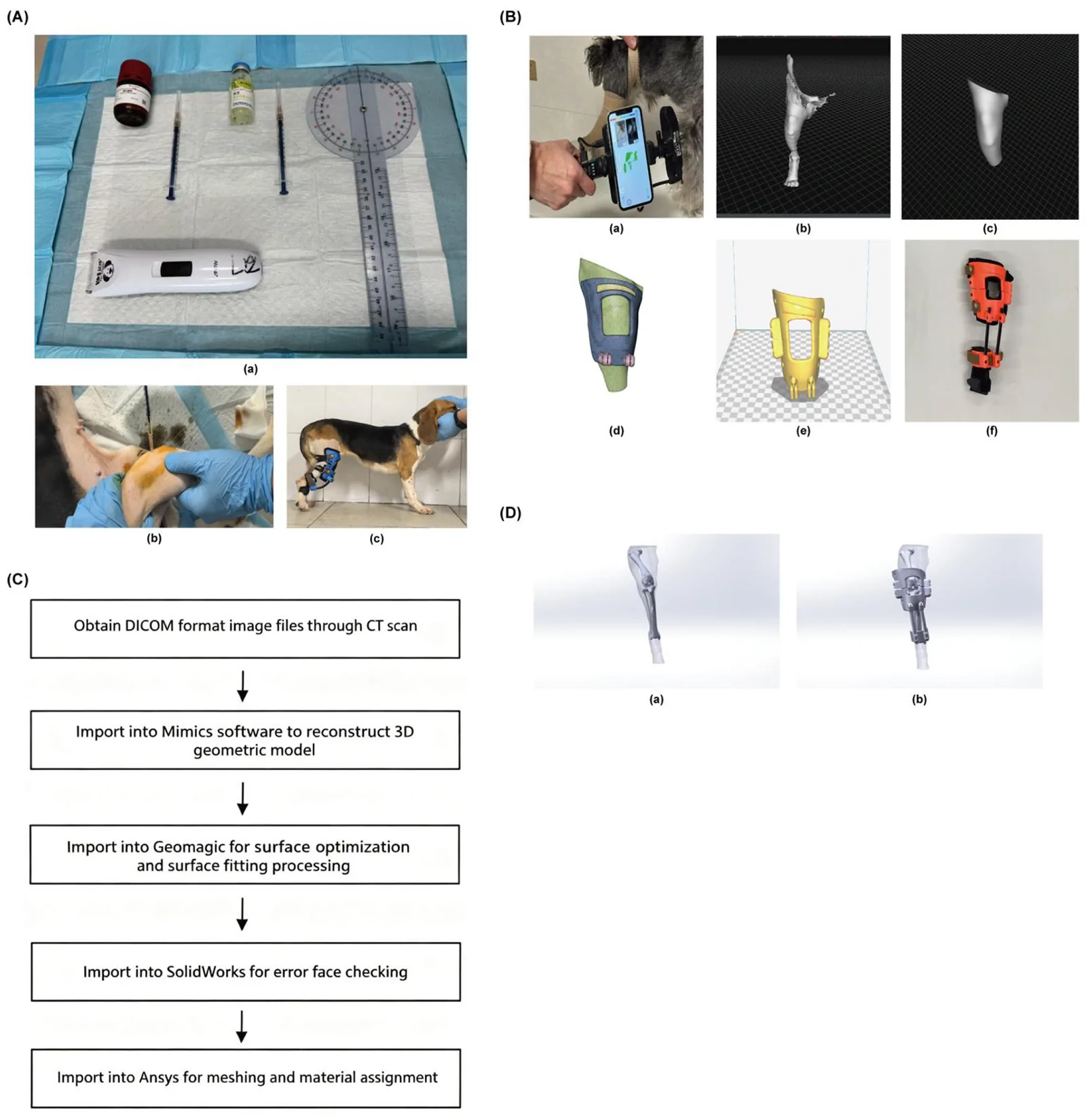
Construction of the canine knee osteoarthritis model, fabrication of the personalized knee splint, and finite element modeling. **(A)** Construction of the arthritis animal model and splint wearing condition: **(a)** main reagents and instruments used in the *in vivo* animal experiment; **(b)** schematic of arthritis animal model construction; **(c)** canine knee splint wearing condition. **(B)** Digital design and fabrication workflow of the personalized 3D-printed canine knee splint: **(a)** three-dimensional surface scanning; **(b)** three-dimensional surface model of the canine hindlimb; **(c)** optimized local knee model; **(d)** personalized digital splint model; **(e)** splint slicing and printing preview; **(f)** final splint after printing, polishing, and assembly. **(C)** Workflow for constructing the canine knee finite element model based on CBCT images. **(D)** Canine knee model and assembled model after splint application.

#### 2.2.2 Measurement tools

Cone-beam computed tomography (CBCT) imaging was performed at the Teaching Animal Hospital of South China Agricultural University. Surface anatomy was captured using a CR-Scan Ferret Pro 3D scanner (Creality 3D, Shenzhen, China). Knee range of motion was measured with a transparent plastic goniometer, and plantar pressure data were recorded using a Sensor Medica pressure-mapping system (Sensor Medica Srl, Rome, Italy).

#### 2.2.3 Clinical scoring tools

LOAD, CBPI, and COI were selected because they are commonly used clinical metrology instruments for evaluating pain-related functional impairment, chronic pain-related behavioral changes, quality-of-life-related outcomes, and treatment response in dogs with OA (18–22). Liverpool Osteoarthritis in Dogs (LOAD) is a 13-item owner-reported instrument that assesses pain-related functional impairment in dogs with OA. Item scores are summed to give a total score from 0 to 52, with higher scores indicating greater impairment (19).

The Canine Brief Pain Inventory (CBPI) includes a Pain Severity Score (PSS; questions 1–4, scored from 0 to 10 and averaged) and a Pain Interference Score (PIS; questions 5–10, scored from 0 to 10 and averaged); higher scores indicate greater pain or functional interference (21, 22).

The Canine Orthopedic Index (COI) subdomains used in this study were stiffness (SD; questions 1–4, total score 0–16), function (FD; questions 5–8, total score 0–16), and gait (GD; questions 9–13, total score 0–20) (20, 23).

Because the experimental dogs were managed under standardized housing conditions, the cold-stimulation item in LOAD was assigned a score of 0, and the COI item regarding how often the disease was noticed was scored as “frequently.” The quality-of-life items in COI (questions 14–16) were not included in the figure analysis.

#### 2.2.4 Finite element modeling

Finite element modeling and analysis were performed using Mimics 21.0, Geomagic Wrap 2021, SolidWorks 2023, and ANSYS Workbench 23.0. These software packages were used to construct digital models, design the splint, and simulate the biomechanical responses of the knee joint and splint.

#### 2.2.5 Data processing and statistical analysis

Data were processed and analyzed using Microsoft Excel, Prism 11, Origin 2026, and RStudio. In the *in vivo* experiment, comparisons among groups were performed using one-way analysis of variance (ANOVA) or two-way ANOVA according to the structure of each outcome, followed by the appropriate multiple-comparison tests. Results are reported as mean ± standard deviation, and *P* < 0.05 was considered statistically significant. No formal interaction analysis was performed; therefore, the combined-treatment results were not interpreted as evidence of an interaction effect.

Finite element analysis was based on representative healthy and OA models and was used as a descriptive mechanical analysis. No group-level statistical testing was performed for the FEA results.

### 2.3 Experimental model induction

Following the modeling protocol of Pomonis et al. (17), all dogs received an intra-articular injection of MIA into the right knee to induce OA. Under sedation, each dog was placed in lateral recumbency, and the right knee region was prepared aseptically. After intra-articular needle placement was confirmed, 100 mg MIA dissolved in 0.5 mL saline was injected near the cruciate-ligament attachment on the medial tibial plateau (Figure 1Ab). The injection was repeated once on the following day.

To control acute inflammatory pain, all dogs received subcutaneous meloxicam for 5 consecutive days beginning on the second day after the first MIA injection. Meloxicam was administered at 0.1 mg/kg, with a doubled first dose of 0.2 mg/kg. From day 7 onward, meloxicam was continued only in the OA/NSAIDs and OA/NSAIDs/Spt groups; treatment was stopped in the OA and OA/Spt groups.

On day 14, successful model induction was clinically defined by persistent lameness and reduced weight-bearing of the affected limb in the absence of obvious knee swelling. Dogs then continued with their assigned interventions.

During the intervention period, dogs in the OA/Spt and OA/NSAIDs/Spt groups continuously wore the personalized 3D-printed PLA knee splint (Figure 1Ac). The skin beneath the splint was checked daily, and the splint was adjusted or temporarily removed if redness or skin injury occurred. Dogs in the OA/NSAIDs and OA/NSAIDs/Spt groups continued to receive meloxicam according to the study protocol, whereas the OA group received no further intervention after the common analgesic period.

Fourteen days after model induction, static weight-bearing assessment, knee range-of-motion measurement, and LOAD, COI, and CBPI scoring were repeated. CBCT images were then acquired under sedation for finite element modeling and structural evaluation.

### 2.4 Scanning, design, and fabrication of the personalized 3D-printed knee splint

The external knee splint was designed and fabricated as follows. Surface anatomical information of the canine knee was captured using a CR-Scan Ferret Pro 3D scanner (Creality 3D; stated accuracy, 0.1 mm) and exported in OBJ format (Figure 1Ba). The acquired three-dimensional knee model was optimized in Geomagic Studio 12 by removing artifacts and smoothing the surface (Figures 1Bb, c). The optimized knee model was then imported into SolidWorks for further splint design, assembly, and trimming, generating the complete external splint model (Figure 1Bd). The splint was fabricated using fused deposition modeling (FDM) 3D printing with a layer height of 0.2 mm and an infill density of 100% (Figure 1Be). A Geeetech A8L 3D printer was used, PLA was selected as the printing material, and the forming accuracy was 0.1 mm. After printing, the components were further polished and assembled to obtain the knee splint (Figure 1Bf). Splint design and printing were completed by trained personnel at Foshan Zhengheng Biotechnology Co., Ltd., Foshan, China.

### 2.5 Finite element analysis

The modeling workflow is shown in Figure 1C. Briefly, knee CBCT data were acquired at a slice thickness of 0.4125 mm and imported into Mimics 21.0. The femur, tibia, fibula, cancellous bone, articular cartilage, and skin were segmented using Hounsfield-unit thresholds, region growing, hole filling, and mask solidification, and were then exported as STL files.

The models were remeshed, smoothed, denoised, and surface-fitted in Geomagic 2021 before being exported as STP files. Bone assembly, interference checks, model repair, and PLA splint construction were completed in SolidWorks 2023. The assembled models were imported into ANSYS Workbench 23.0 in X_T format for static structural analysis.

A tetrahedral mesh was used, and mesh quality was optimized to improve computational stability. All tissues were simplified as isotropic linear elastic materials. Mechanical property parameters are listed in Table 1. A fixed constraint was applied to the distal skin. According to the literature, the hindlimbs of dogs support 39.6% of total body weight (24); therefore, the vertical load on the right hindlimb was approximately 19.8% of body weight. In this study, a vertical load of 21.34 N (11 kg × 9.8 m/s^2^ × 19.8%) was applied proximally to simulate physiological standing. Equivalent von Mises stress, equivalent elastic strain, contact pressure, and total deformation were extracted after solution. Contour maps for all simulated conditions were displayed using a unified color scale. The model schematic is shown in Figures 1Da, b, and the finite element analysis schematic is shown in Figures 1Ea, b. This FEA was used only for descriptive comparison of representative models and does not represent group-level statistical inference (15).

**Table 1:** Mechanical properties of model materials.

Material/tissue	Young's modulus (MPa)	Poisson's ratio
Skin	25 (52, 53)	0.45 (52, 54)
Femur (cortical)	14,560 (55–57)	0.30 (56, 58)
Femoral cancellous bone	100 (59, 60)	0.20 (61)
Tibia (cortical)	13,880 (57, 58)	0.30 (58, 61)
Tibial cancellous bone	120 (62)	0.25 (63)
Fibula (cortical)	14,000 (57, 61)	0.40 (64)
Fibular cancellous bone	700 (61)	0.20 (61)
Splint (PLA)	3,000 (65, 66)	0.35 (66)
Articular cartilage	1 (67, 68)	0.46 (69)

### 2.6 *In vivo* experiment

#### 2.6.1 Static hindlimb weight-bearing assessment

Each dog was allowed to acclimate to the testing area for at least 5 min and was trained to stand quietly with the affected limb positioned within the active detection area of the pressure platform. Body position was kept as consistent as possible across tests. During each test, 4 s of static ground-reaction-force data, motion-capture data, and synchronized images were recorded. A trial was included in the analysis only when the dog remained stationary throughout the recording period. Peak vertical force was then extracted for subsequent analysis (25, 26).

The hindlimb weight-bearing symmetry index (SI) was calculated as follows:

$$\begin{array}{l} \text{SI}=\left[\frac{1}{2}\times \frac{\left(X_{R}-X_{L}\right)}{\left(X_{R}+X_{L}\right)}\right]\times 100\% \end{array}$$ (1)

Where XR is the selected ground reaction force (GRF) for the right hindlimb and XL is the corresponding value for the left hindlimb. SI ranges from −50 to +50, with 0 indicating equal bilateral loading (27).

### 2.7 Knee joint range of motion (ROM) measurement

Knee flexion-extension range of motion was measured with a transparent plastic goniometer. Measurements were obtained in a quiet, warm environment while the dog was relaxed; mild sedation was used when necessary (28).

With the dog in lateral recumbency and the tested limb uppermost, the goniometer axis was placed over the lateral femoral condyle. The stationary arm was aligned with the greater trochanter and the moving arm with the lateral malleolus. The knee was gently extended to the maximum pain-free position and then flexed to its maximum range. The same operator performed all measurements using the same anatomical landmarks. Each angle was measured three times, and the mean was used for analysis (29).

### 2.8 Clinical pain and function scoring

Pain and function were assessed using LOAD, COI, and CBPI. The instruments were completed by six trained staff members at Guangzhou Liming Biotechnology Co., Ltd. after scoring training provided by relevant teachers from the College of Veterinary Medicine, South China Agricultural University. At fixed time points, each staff member scored two beagles three times, and the result was calculated as the mean value. These staff members participated in the dogs' daily care and were familiar with their usual behavior. Assessors followed the scoring rules for each instrument, and the results were used for subsequent statistical analysis.

Assessments were completed before model induction and again on day 14 after model induction. Assessors were not given the results of the objective outcome measurements. Group masking was attempted during scoring, although complete masking could not be guaranteed because staff were involved in daily animal care and splint use was visible.

## 3 Results

### 3.1 Finite element analysis results

To investigate the biomechanical effects of the knee splint on the beagle knee joint under different simulated conditions, representative finite element models were first used to evaluate the mechanical stability of the personalized 3D-printed knee splint and its effects on mechanical load distribution in canine knee bone and cartilage structures. The finite element analysis was used to provide descriptive, model-based mechanical evidence, and no group-level statistical testing was performed.

#### 3.1.1 The splint remained mechanically stable under simulated loading and reduced peak von Mises stress in bone and cartilage structures in the OA model

To first determine whether the splint could tolerate the simulated loading condition and alter stress distribution in the knee model, the mechanical response of the splint itself and the von Mises stress distribution of knee bone and cartilage structures were analyzed together in Figure 2. Under the simulated loading condition, the splint showed limited overall deformation, with a maximum equivalent elastic strain of 0.0043 (0.43%). After the splint was assembled on the canine knee model, stress was mainly concentrated at the support rods and connecting regions, and the maximum equivalent stress was 12.87 MPa, which was below the reported yield strength of PLA (40 MPa). This result suggests that, under the simulated standing load used in this study, the splint structure remained below the material yield threshold and showed basic mechanical stability (Figure 2A).

**Figure 2:**
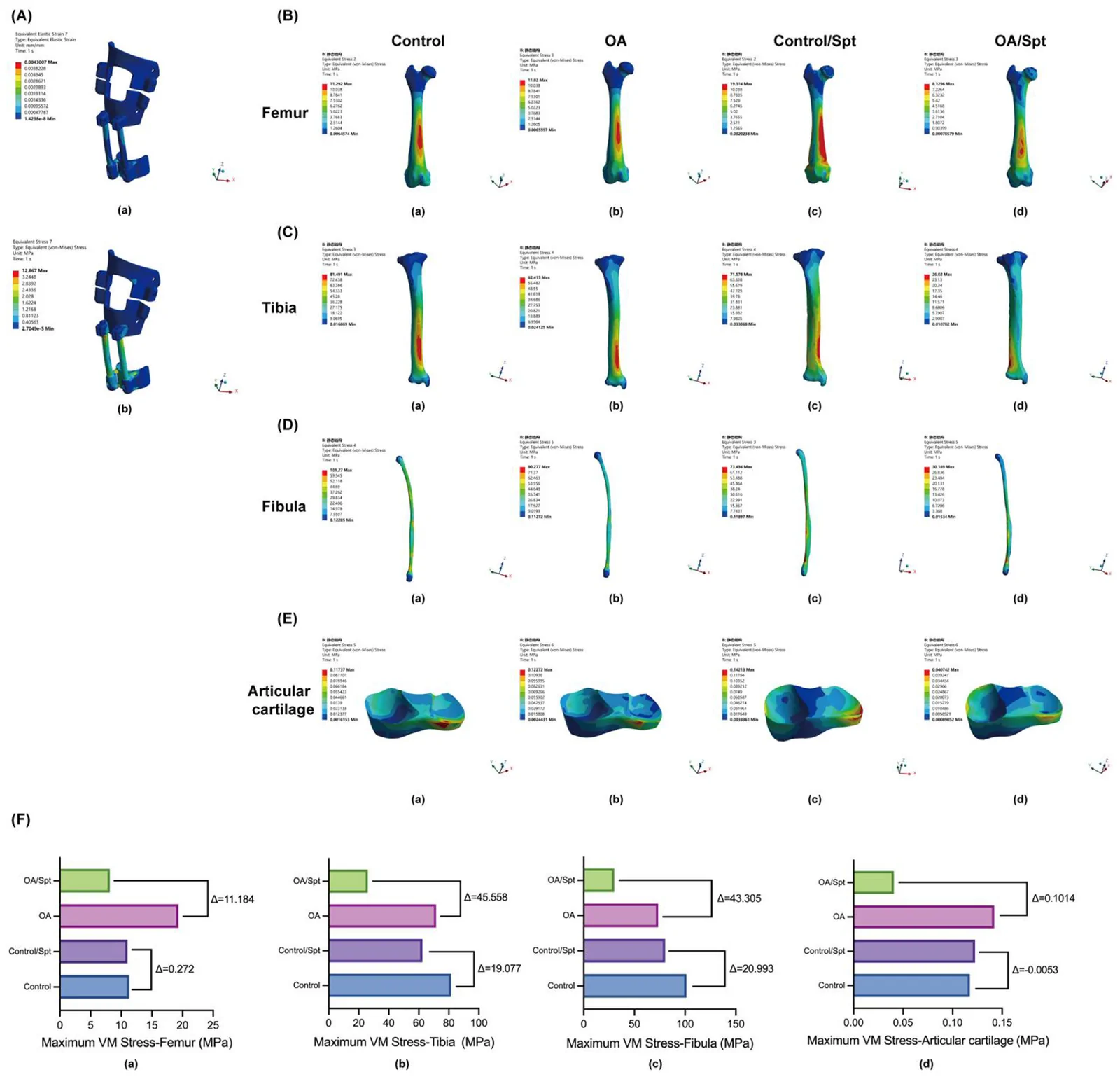
Mechanical distribution of the knee splint and von Mises stress distribution in canine knee bone and cartilage structures under different simulated conditions. **(A)** Equivalent elastic strain and equivalent stress distributions of the knee splint under simulated loading. **(B)** Femur, **(C)** tibia, **(D)** fibula, and **(E)** articular cartilage von Mises stress contour maps under Control, OA, Control/Spt, and OA/Spt conditions. **(F)** Bar graph comparison of peak von Mises stress in each structure. Δ indicates the difference in peak stress before and after splint application within the same model.

To further compare how splint application affected bone and cartilage stress under healthy and OA conditions, von Mises stress was examined under Control, OA, Control/Spt, and OA/Spt conditions. High-stress regions in the femur, tibia, fibula, and articular cartilage were mainly distributed in the diaphyses and weight-bearing articular surfaces (Figures 2B–E). After splint application in the healthy control model, peak von Mises stress decreased by 0.272, 19.077, and 20.993 MPa in the femur, tibia, and fibula, respectively, whereas peak articular cartilage stress increased slightly by 0.005 MPa (Figure 2F). This result suggests that, in the healthy model, the splint showed a degree of unloading tendency in bone structures but did not reduce peak cartilage stress.

Compared with the healthy control model, the OA model showed higher peak stress in the tibia and fibula and more obvious high-stress regions. After splint application in the OA model, peak von Mises stress decreased by 11.184, 45.558, 43.305, and 0.101 MPa in the femur, tibia, fibula, and articular cartilage, respectively (Figure 2F). Compared with the healthy model, the OA model showed larger reductions in peak stress after splint application, suggesting that the splint may have greater mechanical unloading potential under the OA condition. Importantly, the results in Figure 2 were derived from representative finite element models and were intended to support the mechanical plausibility and potential unloading effect of the splint, rather than to serve as *in vivo* evidence of therapeutic efficacy.

#### 3.1.2 The splint reduced femoral condylar contact pressure and cartilage total deformation in the OA model

To examine whether splint application could reduce localized articular loading at the femoral condyles, femoral condylar contact pressure was compared across the four simulated conditions. The healthy control model had a relatively even contact-pressure distribution, with a peak contact pressure of 0.1861 MPa. After splint application, the value decreased to 0.1602 MPa, a reduction of 0.0259 MPa (Figures 3A, C). In the OA model, the high-pressure region was concentrated toward the medial condyle, and the peak contact pressure was 0.2384 MPa. After splint application, the value decreased to 0.0753 MPa, a reduction of 0.1631 MPa (Figures 3A, C). The reduction in contact pressure in the OA model was approximately 6.3-fold greater than that in the healthy model, suggesting that the splint may more clearly reduce localized articular contact pressure in the OA model.

**Figure 3:**
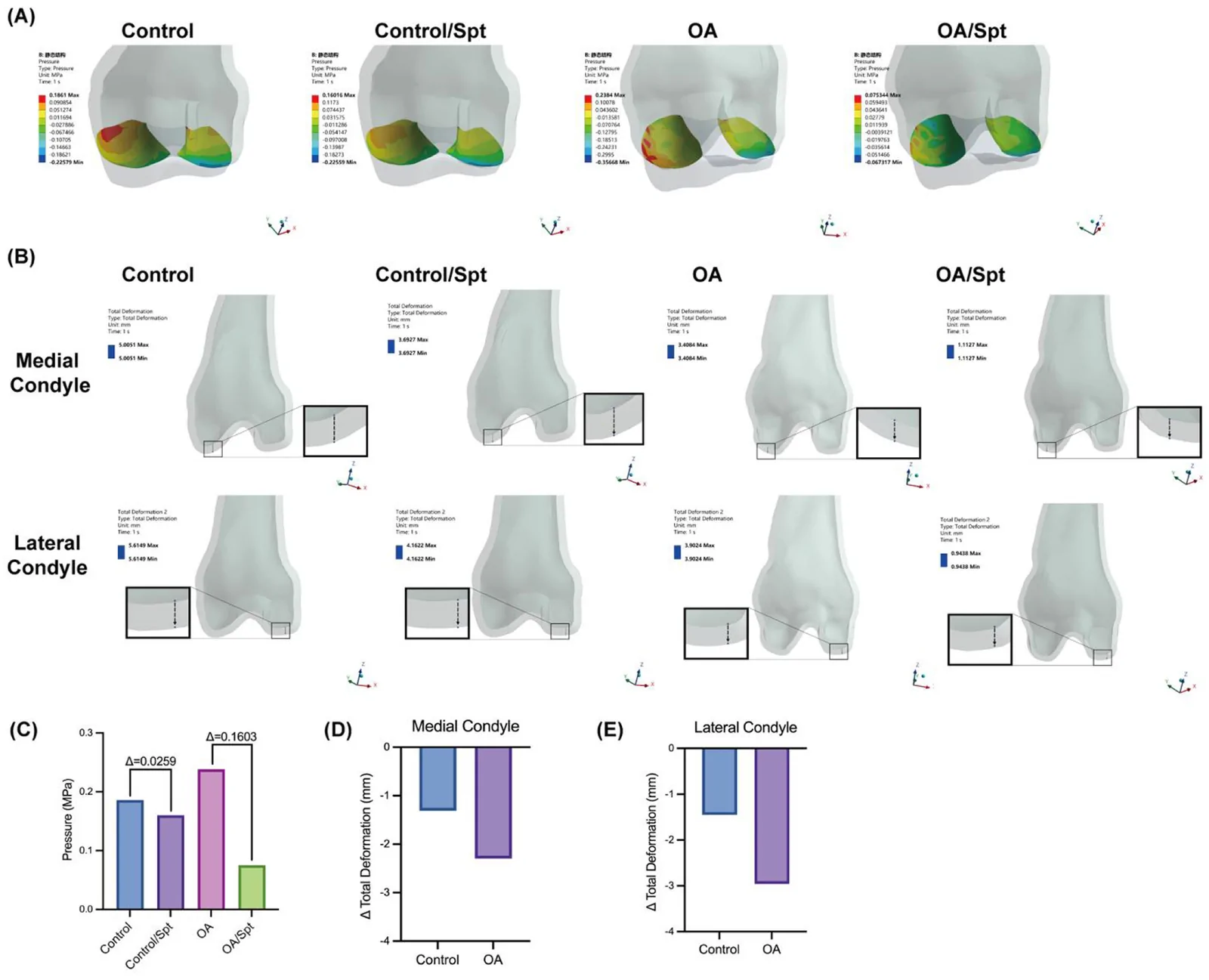
Changes in femoral condylar articular contact pressure and cartilage total deformation under different simulated conditions. **(A)** Femoral condylar articular contact-pressure distribution under Control, Control/Spt, OA, and OA/Spt conditions. **(B)** Total deformation distribution of medial and lateral femoral condylar cartilage under different simulated conditions. **(C)** Bar graph comparison of peak femoral condylar contact pressure. Δ indicates the difference in peak contact pressure before and after splint application within the same model. **(D, E)** Changes in maximum total deformation of the medial and lateral femoral condyles after splint application.

Analysis of cartilage total deformation showed that total deformation was mainly concentrated at the weight-bearing contact surface of the femoral condyle (Figure 3B). In the healthy control model, after splint application, maximum total deformation decreased from 5.0051 to 3.6927 mm in the medial condyle and from 5.6149 to 4.1622 mm in the lateral condyle, with reductions of 1.3124 and 1.4527 mm, respectively (Figures 3D, E). In the OA model, after splint application, maximum total deformation decreased from 3.4048 to 1.1127 mm in the medial condyle and from 3.9024 to 0.9438 mm in the lateral condyle, with reductions of 2.2921 and 2.9586 mm, respectively (Figures 3D, E). These results indicate that the splint reduced model-predicted cartilage total deformation in both models, with larger reductions in the OA model. Because the unsplinted OA model did not show greater baseline cartilage total deformation than the unsplinted healthy control model, this result should be interpreted as a model-predicted change before and after splint application, rather than evidence that OA itself increased cartilage deformation.

#### 3.1.3 The splint reduced equivalent elastic strain in multiple knee structures in the OA model

To evaluate whether the splint also influenced tissue strain, equivalent elastic strain was compared in the femur. Equivalent elastic strain contour maps showed that high-strain regions were mainly located at the epiphyses and weight-bearing cartilage surfaces (Figures 4A–D). Without splint application, the OA model showed higher peak equivalent elastic strain and more obvious high-strain regions than the healthy control model. As shown in Table 2. After splint application in the healthy control model, peak equivalent elastic strain decreased by 20.25%, 30.86%, and 26.21% in the femur, tibia, and fibula, respectively, whereas peak articular cartilage strain increased slightly by 4.55%. After splint application in the OA model, peak equivalent elastic strain decreased by 80.46%, 76.57%, 72.90%, and 71.54% in the femur, tibia, fibula, and articular cartilage, respectively. Thus, the splint showed more obvious strain reduction in both bone structures and articular cartilage in the OA model.

**Figure 4:**
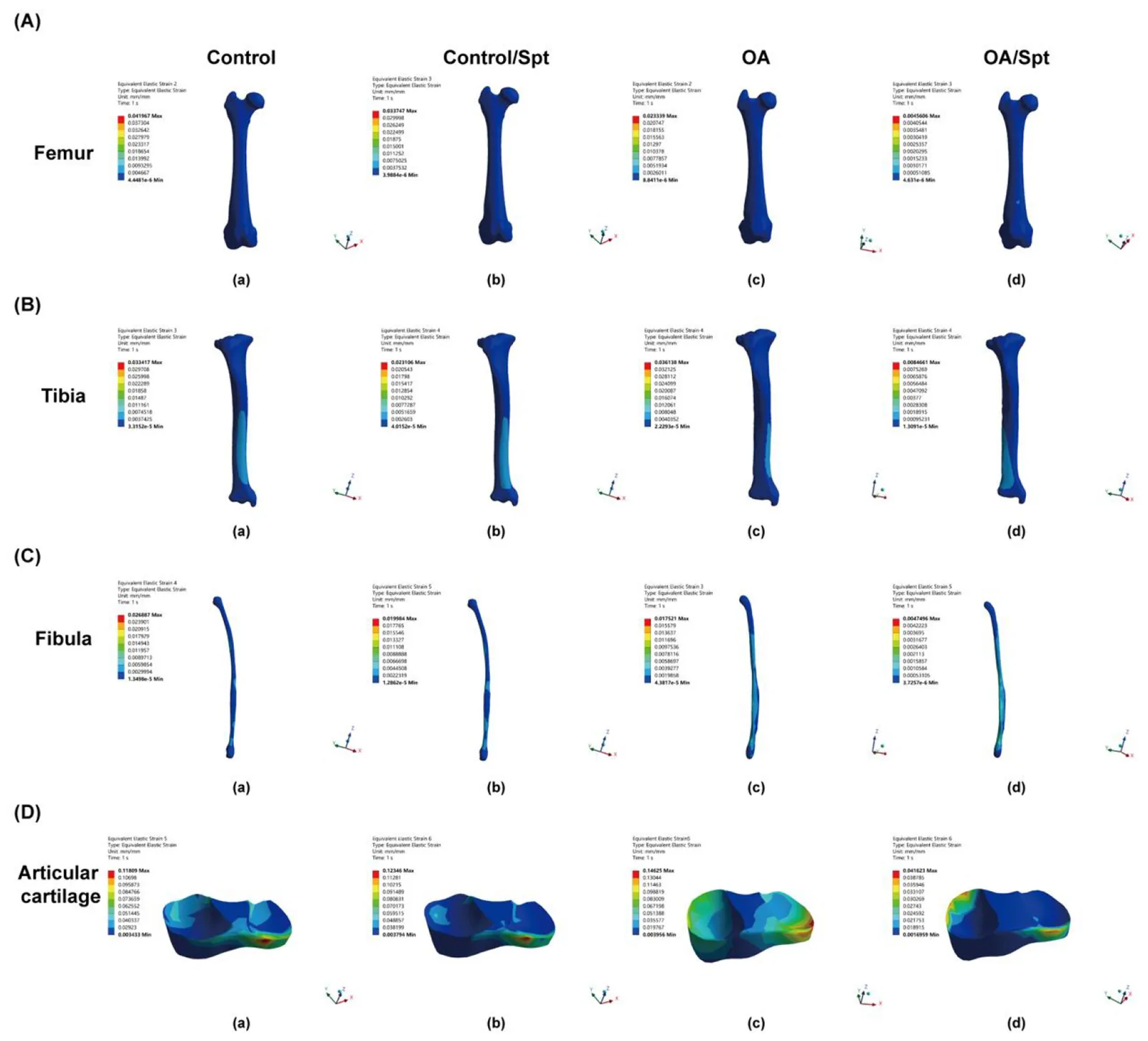
Equivalent elastic strain distribution in canine knee bone and cartilage structures under different simulated conditions. **(A)** Femur, **(B)** tibia, **(C)** fibula, and **(D)** articular cartilage equivalent elastic strain contour maps under Control, Control/Spt, OA, and OA/Spt conditions. These finite element results represent a descriptive analysis of representative models and support the mechanical plausibility of the splint.

**Table 2:** Reduction rate of peak equivalent elastic strain in knee joint structures after splint intervention.

Structure	Control vs. control/spt reduction rate (%)	OA vs. OA/spt reduction rate (%)
Femur	20.25%	80.46%
Tibia	30.86%	76.57%
Fibula	26.21%	72.90%
Articular cartilage	−4.55%	71.54%

### 3.2 *In vivo* functional outcomes

To further evaluate the effects of the knee splint and nonsteroidal anti-inflammatory drugs on short-term functional outcomes in the MIA-induced canine knee OA model, we assessed *in vivo* outcomes including static hindlimb weight-bearing symmetry, knee range of motion, and LOAD, CBPI, and COI pain and function scores.

#### 3.2.1 Splint and NSAID treatment improved hindlimb weight-bearing symmetry, and the combined-treatment group was closest to symmetrical loading

To determine whether each intervention improved affected-limb loading, static hindlimb weight-bearing symmetry was assessed before model induction and after intervention. The apparatus and procedure used for static weight-bearing assessment are shown in Figures 5Aa, b. Before model induction, SI values were close to 0 in all groups, indicating relatively symmetrical hindlimb loading. After model induction and intervention, the OA group showed the most obvious loading asymmetry, whereas SI values in the OA/Spt and OA/NSAIDs groups shifted closer to 0. And significantly differences in SI were observed among the groups after intervention. The combined-treatment group differed significantly from the OA, OA/Spt, and OA/NSAIDs groups. The OA/NSAIDs/Spt group was closest to symmetrical loading after intervention (Figures 5B, C).

**Figure 5:**
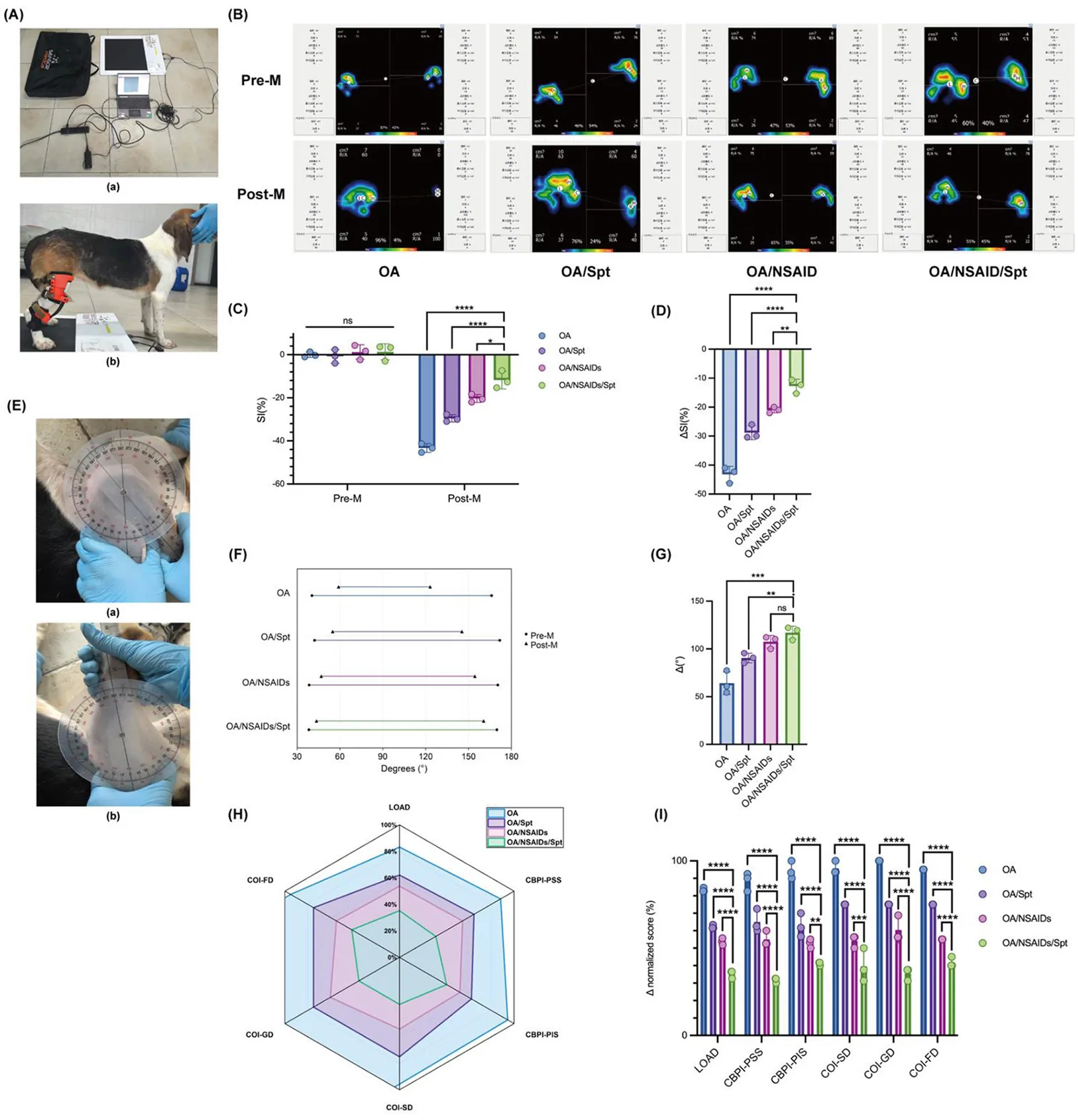
*In vivo* functional outcomes after short-term combined intervention with the knee splint and nonsteroidal anti-inflammatory drugs. **(A)** Static plantar pressure detection system and canine static weight-bearing test procedure: **(a)** static plantar pressure detection system; **(b)** schematic of static pressure testing in beagles. **(B)** Representative plantar pressure images of each group before model induction and after intervention. **(C)** Changes in hindlimb weight-bearing symmetry index (SI) before model induction and after intervention in each group. **(D)** Changes in SI (ΔSI) in each group. **(E)** Schematic of ROM testing in beagles: **(a)** measurement during joint flexion; **(b)** measurement during joint extension. **(F)** Changes in knee range of motion (ROM) before model induction and after intervention in each group. **(G)** Changes in ROM (ΔROM) in each group. **(H)** Radar plot of post-intervention normalized scores for LOAD, CBPI-PSS, CBPI-PIS, COI-SD, COI-GD, and COI-FD. The normalized score was calculated as actual score / maximum possible score × 100%. **(I)** Changes in normalized scores for each scale. Δ normalized score was calculated as post-intervention normalized score minus pre-modeling normalized score. Lower post-intervention scores or smaller score increases indicate less pain or functional impairment. Data are presented as mean ± SD (*n* = 3 independent samples), with individual data points shown. Two-way ANOVA was used for **(C)** and **(I)**, and one-way ANOVA was used for **(D)** and **(G)**. ns, not significant; *p < 0.05; **p < 0.01; ***p < 0.001; ****p < 0.0001.

Analysis of ΔSI further supported this pattern. The combined-treatment group showed a greater shift toward balanced loading than the OA group and was also closer to symmetry than the NSAID-alone group (Figure 5D). These findings suggest that splint treatment and NSAID treatment each contributed to improved short-term limb use, with the combined group showing the most favorable static weight-bearing profile.

#### 3.2.2 The combined-treatment group showed the smallest ROM decrease

To assess joint mobility after model induction and intervention, knee ROM was measured in each group. The measurement procedure is illustrated in Figure 5E. Before model induction, affected-limb ROM was close to contralateral-limb ROM in each group, indicating relatively comparable baseline joint range of motion. After model induction and intervention, affected-knee ROM decreased in all groups, but the magnitude of decrease differed among groups (Figure 5F). The OA group showed the largest ROM decrease, with a mean ΔROM of −61.3 ± 5.1°. Mean ΔROM values in the OA/Spt, OA/NSAIDs, and OA/NSAIDs/Spt groups were −39.2 ± 2.9°, −24.6 ± 3.5°, and −14.6 ± 3.2°, respectively (Figure 5G; Table 3). As shown in Figure 5G, the OA/NSAIDs/Spt group did not differ significantly from the OA/NSAIDs group, but showed significant differences compared with the OA and OA/Spt groups. The results presented in Table 3 further showed that the within-group comparisons among individual dogs were not statistically significant in any group (all *P* > 0.05).

**Table 3:** Joint range of motion (ROM) measurements in beagle dogs of each group (Unit: °, Mean ± SD).

Group	Pre-modeling (pre-M)		Post-modeling ROM (post-M)	ΔROM (post-M - pre-M)	Intragroup significance
	Affected leg (right)	Unaffected leg (left, control)			
OA	125.8 ± 3.9	126.1 ± 3.4	64.2 ± 11.8	−61.3 ± 5.1	ns (*P* > 0.05)
OA/Spt	129.7 ± 4.9	130.5 ± 3.7	90.5 ± 5.1	−39.2 ± 2.9	ns (*P* > 0.05)
OA/NSAIDs	132.1 ± 2.7	131.1 ± 2.4	107.5 ± 6.5	−24.6 ± 3.5	ns (*P* > 0.05)
OA/NSAIDs/Spt	131.6 ± 9.3	132.4 ± 7.7	117.1 ± 6.8	−14.6 ± 3.2	ns (*P* > 0.05)

ns, not significant.

These results indicate that splint treatment alone and NSAID treatment alone were both associated with smaller ROM decreases. NSAID treatment alone showed a more obvious short-term improvement than splint treatment alone, and the combined-treatment group showed the smallest ROM decrease. This result supports better short-term joint mobility after combined splint plus NSAID intervention, but it does not by itself demonstrate an interaction effect between the two interventions.

#### 3.2.3 The combined-treatment group showed the lowest post-intervention normalized scores, and smaller score increases for pain and function assessments

To compare pain and function outcomes across scoring instruments with different maximum scores, LOAD, CBPI-PSS, CBPI-PIS, COI-SD, COI-GD, and COI-FD were normalized by the maximum possible score of each scale or domain. The radar plot of post-intervention normalized scores showed that the OA group had higher scores in most scoring items, indicating more obvious pain and functional impairment. Scores in the OA/Spt and OA/NSAIDs groups were lower than those in the OA group, and the NSAID-alone group was generally lower than the splint-alone group. The OA/NSAIDs/Spt group showed the lowest scores in most scoring items (Figure 5H).

To evaluate score changes from baseline, Δ normalized scores were then calculated as post-intervention normalized score minus pre-modeling normalized score. The Δ normalized score results showed differences in score increases among groups and scoring items (Figure 5I). Overall, the OA group showed the largest score increases, the OA/Spt group showed an intermediate improvement, the OA/NSAIDs group showed further reduced score increases, and the OA/NSAIDs/Spt group showed smaller score increases in most scoring items. Lower post-intervention normalized scores or smaller score increases indicate less pain or functional impairment. These results suggest that combined splint plus NSAID intervention provided broader short-term functional benefit across multiple pain and function scoring outcomes.

## 4 Discussion

This study evaluated a personalized 3D-printed knee splint as a mechanical adjunct to NSAID therapy in a canine knee OA model. At the biomechanical level, FEA indicated that the splint was mechanically stable and reduced model-predicted stress, strain, contact pressure, and cartilage deformation in a representative OA model. At the functional level, the *in vivo* findings showed a similar pattern: splint treatment alone was associated with short-term functional improvement compared with no continued treatment, NSAID treatment produced a stronger response, and the combined OA/NSAIDs/Spt group showed the most consistent improvement across weight-bearing symmetry, ROM, and pain/function scores.

### 4.1 Abnormal mechanical loading and OA pain-related dysfunction

OA pain and dysfunction do not arise only from inflammation. Abnormal joint mechanics can alter chondrocyte mechanotransduction, affect Wnt-integrin signaling, mechanosensitive ion channels, and YAP/TAZ (Yes-associated protein/transcriptional coactivator with PDZ-binding motif)-related pathways, and promote inflammatory mediator release and extracellular matrix degradation (30–33). Increased mechanical stimulation of cartilage, subchondral bone, capsule, ligaments, synovium, and the osteochondral interface may contribute to protective unloading, reduced activity, and postural adjustment (34–36).

The finite element results fit this framework: the OA model showed stress or strain concentration in bone and cartilage and application of splint reduced some mechanical indices (particularly in the OA model). The FEA provides two related but distinct types of mechanical information: first, the maximum stress in PLA remained below the reported material yield strength under the standing load, suggesting that the device was still in the elastic range under this load. Second, the application of splint decreased several model-predicted mechanical indices in the representative OA model, including bone and cartilage stress, equivalent elastic strain, femoral condylar contact pressure and cartilage total deformation. These results support the mechanical plausibility of the splint as a possible external unloader.

### 4.2 How mechanical unloading may translate into pain-related functional improvement

OA pain is not determined only by visible structural damage; it also involves nociceptor activation, inflammatory mediators, mechanical stimulation, and peripheral or central sensitization (36–38). By reducing model-predicted stress, strain, and contact pressure, the splint may lessen abnormal mechanical stimulation during weight bearing. This could provide one possible explanation for more balanced loading and better preservation of knee motion.

The SI and ROM results are consistent with this interpretation. SI values closer to 0 indicate more balanced static loading between limbs, whereas a smaller ROM decrease may reflect less restriction from pain, stiffness, or protective muscle tension (39, 40). The combined-treatment group had the most symmetrical SI profile and the smallest ROM decrease, indicating the most favorable short-term pattern of weight bearing and knee mobility among the four groups. The improvement in static hindlimb weight-bearing symmetry was consistent with more balanced affected-limb loading during quiet standing. Because the present assessment focused on static loading rather than dynamic gait, these findings should not be interpreted as evidence of improved walking mechanics. Effects on stride length, stance time, peak vertical force during walking, and other dynamic gait parameters remain to be determined using quantitative gait analysis (41).

### 4.3 Complementary effects of the splint and NSAIDs

NSAIDs reduce prostaglandin production through cyclooxygenase inhibition and thereby reduce inflammation and pain (42, 43). This may improve tolerance for loading and movement, but it does not directly correct abnormal joint mechanics. The splint provides a different contribution: external support and local load redistribution during limb use.

The distinct proposed roles of NSAIDs and splinting may help explain the pattern observed in the combined-treatment group. Pharmacological pain control may make dogs more willing to use the affected limb, while mechanical support may allow the limb to bear weight with less abnormal stimulation. Thus, the combined group showed broader short-term functional benefit across SI, ROM, and clinical score domains. Because no formal interaction analysis was performed, these findings are described as complementary benefit (44–47).

### 4.4 Functional meaning of the clinical score improvements

Functional improvement was supported by objective loading, joint mobility, and multidimensional clinical scores. LOAD reflects overall activity and pain-related functional impairment. CBPI-PSS reflects pain severity, and CBPI-PIS reflects pain interference with activity and daily life. COI-SD, COI-FD, and COI-GD correspond to stiffness, function, and gait; lower scores indicate less stiffness, better movement ability, and less gait abnormality (48).

Across the objective and clinical measures, the combined-treatment group showed the most favorable short-term functional profile. It had the smallest ROM decrease (−14.6 ± 3.2°), the highest post-intervention ROM (117.1 ± 6.8°), and the SI value closest to symmetrical loading. These findings were consistent with the lower post-intervention scores or smaller score increases observed across most LOAD, CBPI, and COI domains.

### 4.5 Clinical implications and translational potential

A personalized 3D-printed splint converts anatomical information from scanning or imaging into an external support fitted to the affected limb. Compared with a standardized brace, a patient-specific design may better match limb contour and facilitate targeted mechanical support. However, these potential advantages require direct comparison with standardized devices and longer-term assessment of comfort, skin tolerance, and mobility (49, 50).

Its potential translational value lies in its possible use as a complement to, rather than a replacement for, NSAID therapy. If future studies confirm benefits in naturally occurring OA, different breeds, and longer follow-up, personalized splinting may become a practical component of integrated canine OA management.

### 4.6 Limitations and future directions

This study has several limitations. First, each group included only three dogs, and no *a priori* sample-size calculation was performed. Statistical power was therefore limited, and individual variation could influence the results. Second, the observation period was 14 days after model induction and intervention. The MIA model can induce pain-related behavior and joint structural changes within a short period (17, 51), making it useful for studying early or short-term intervention responses. However, a 14-day follow-up is not sufficient to evaluate long-term efficacy, chronic pain evolution, or disease modification. Third, the finite element analysis was based on representative models. Material properties and boundary conditions were necessarily simplified, and the results cannot replace subject-specific experimental measurements or population-level inference.

Histopathological, structural imaging, inflammatory, and molecular endpoints were not included. Therefore, the observed improvements in SI, ROM, and clinical scores cannot be interpreted as direct evidence of cartilage protection, synovial improvement, or delayed structural progression. Larger studies with longer follow-up should incorporate subject-specific finite element models, mechanical validation, structural imaging, histopathology, inflammatory markers, and pain-related neurobiological endpoints.

## 5 Conclusions

In this preliminary short-term canine knee OA model, the personalized 3D-printed splint was mechanically stable under simulated loading and reduced model-predicted mechanical indices in a representative OA knee model.

*In vivo*, the OA/NSAIDs/Spt group showed the most favorable overall short-term functional profile in static hindlimb weight-bearing symmetry, ROM, and LOAD, CBPI, and COI pain/function scores, while splint alone and NSAIDs alone each provided partial short-term benefit.

Overall, these findings support personalized 3D-printed splinting as a potential mechanical adjunct to NSAID therapy for canine knee OA. Larger and longer-term studies in different breeds and naturally occurring OA are needed to confirm efficacy, safety, and clinical applicability.

## Data Availability

The raw data supporting the conclusions of this article will be made available by the authors, without undue reservation.
